# The relationship between structural changes in paraspinal muscles and intervertebral disc and facet joint degeneration in the lumbar spine of rats

**DOI:** 10.1186/s13018-024-04548-8

**Published:** 2024-01-12

**Authors:** Artem Skidanov, Nataliya Ashukina, Valentyna Maltseva, Mykyta Skidanov, Zinaida Danyshchuk, Volodymyr Radchenko

**Affiliations:** Laboratory of Connective Tissue Morphology, Sytenko Institute of Spine and Joint Pathology National Academy of Medical Sciences of Ukraine, 80 Pushkinska St., Kharkiv, 61024 Ukraine

**Keywords:** Rats, Annulus fibrosus, Cartilage, Intervertebral disc, Facet joint osteoarthritis, Zygapophyseal joint, Paraspinal muscles, Histology

## Abstract

**Background:**

Degenerative spine disease is one of the largest causes of disability worldwide and has a multifactorial aetiology. Determining the leading causes of this multifactorial disease could help create new treatment approaches.

**Purpose:**

Study the impact of degenerative changes in the paraspinal muscles caused by local (prolonged compression) or systemic (high-fat diet) factors on the structure of the intervertebral discs (IVDs) and facet joints of the lumbar spine in rats.

**Methods:**

The study was conducted using two animal models to create degenerative changes in the paraspinal muscles of 10 white laboratory rats for 90 days and five control rats: 1) high-fat diet model (model 1) involved keeping the rats on a high calorie diet; 2) compression model (model 2) involved binding the paraspinal muscles from L2 to S1 using non-absorbable sutures. Histological analysis for the facet joints and IVDs of rats (at the L1–L4 level) with semi-quantitative analysis of the structure conducted used by degeneration grading system for IVDs and cartilage degeneration score (OARSI) for facet joint.

**Results:**

In both models, 90 days after the experiment, the degenerative changes observed in the rats’ IVDs were more severe in the annulus fibrosus than in the nucleus pulposus. The height of the IVD in model 1 did not differ from the control group, but in the model 2 was 1.3 times greater (*p* < 0.001) compared with control. Degenerative changes in the IVD were scored out 5.3 ± 1.7 in model 1 and 5.32 ± 2.1 in model 2 of a possible 16. The height of the articular cartilage of the facet joints was smaller by 1.5 times (*p* < 0.001) and 1.4 times (*p* < 0.001) in model 1 and model 2, respectively, compared to the control. Degenerative changes of facet joint were scored out 3.7 ± 0.6 in model 1 and 3.8 ± 0.6 in model 2 of five points according to the cartilage degeneration score.

**Conclusions:**

It was determined that rats who had structural changes in the lumbar paraspinal muscles as a result of being kept on a high-fat diet or subjected to prolonged compression for 90 days, showed degenerative changes in intervertebral discs and osteoarthritis in facet joints of lumbar spine.

## Background

Low back pain (LBP) is one of the largest causes of disability worldwide, with approximately 10% of the world’s population suffering from LBP, and that value continues to grow [1]. Degenerative diseases of the spine (osteochondrosis, spondyloarthrosis, etc.) all have a similar multifactorial aetiology [2], which makes it hard to determine the main cause of their development. It is known that pathological changes to the intervertebral disc (IVD) and facet joint, which often cause LBP (45 and 40%, respectively) [3], are affected by age, systemic regulation factors (hormones, peptides, cytokines), genetic predisposition, excessive load, harmful environmental factors, etc. [4].

Recently, researchers have focused on the state of paraspinal muscles (*m. multifidus*, *m. erector spinae, m. quadratus lumborum*, *m. psoas major*) in patients with LBP. This is due to the crucial role of these muscles in ensuring the mechanical stability of the spine and protecting spinal structures from being damaged by excessive loads [5]. Changes in the paraspinal muscles with age, as a result of trauma, or degenerative processes inevitably lead to diminished function, which can result in chronic LBP [6, 7].

It is known that the most common causes of feelings of pain in the lumbar spine include structural changes in the IVD (45%), facet (40%), and sacroiliac joints (13%) [3]. However, there is no conclusive proof of the relationship between the severity of structural changes in the paraspinal muscles and the level of degeneration in the IVD. When experimenting on dogs with chondrodystrophy that spontaneously developed hernia of IVD and lumbar pain, no clear relationship was discovered between fat accumulation in the muscles and the severity of IVD degeneration [8]. Specifically, lower fat content was observed in the paraspinal muscles of dogs with a higher index of IVD degeneration. Earlier, we showed that as a result of keeping rats on a high-fat diet and modelling prolonged compression of the paraspinal muscles, degenerative changes appear in the m. multifidus and m. psoas, similar to those in patients with degenerative spine diseases [9].

Clinical studies obtained contradictory data about the relationship between structural changes in paraspinal muscles and degeneration in the IVD, facet joints, and sacroiliac joints. After analysing MRI scans of 107 patients (age 50.9 ± 15.4 years old), researchers discovered a strong correlation between fatty infiltration of paraspinal muscle and disc degeneration, but a weak correlation with degeneration of facet joints, and a lack of correlation with clinical symptoms, measured using VAS [10]. Kalichman et al. [11] evaluated literary sources that studied patients using imaging methods to describe the features of paraspinal muscles and their relationship with lumbar pain. The authors demonstrated that fatty infiltration in the lumbar multifidus is frequently connected to LBP, especially in women, and is found in the areas with the most severe spinal degeneration. A meta-analysis of 25 works [12] showed a negative correlation between LBP and the area of the cross section of the m. multifidus and discovered contradictory data regarding the effect of fatty infiltration on this muscle. The authors did not find proof of a relationship between fatty infiltration of the m. erector spinae, m. psoas, m. quadratus lumborum, and occurrences of lumbar pain.

The current understanding of the many changes in the morphology and physiology of IVDs did not lead to the creation of a widely accepted model for the development of degenerative spine diseases. This complicates the development of pathogenetic methods of treating degenerative spine diseases [13, 14]. Studying the relationship between the structure and function of the paraspinal muscles and spinal degeneration can help determine the leading causes of the development of this multifactorial disease and the manifestation of lumbar pain, and create new treatment approaches.

*The aim* of this study was to evaluate the impact of degenerative changes in the paraspinal muscles caused by local (prolonged compression) or systemic (high-fat diet) factors on the structure of the intervertebral discs and facet joints of the lumbar spine in rats.

## Methods

### Study design

The experimental study was conducted in accordance with international (Directive 2010/63/EU) and national guidelines on the care and use of laboratory animals. The plan for the experiment was approved by the local bioethics committee (protocol No. 191 from 22.04.2019).

The study was conducted using two models to create degenerative changes in the paraspinal muscles of 10 white male laboratory rats (aged 2 months, weight 140–160 g, five rats in each group).

The *high-fat diet model* (*n* = 5) involved keeping the rats on a high calorie diet, adapted from the Teklad Custom Diet TD.10670 (22.5 g or 40–45% of daily calories from fat) developed by Envigo for laboratory rats or mice [15].

**Fig. 1:**
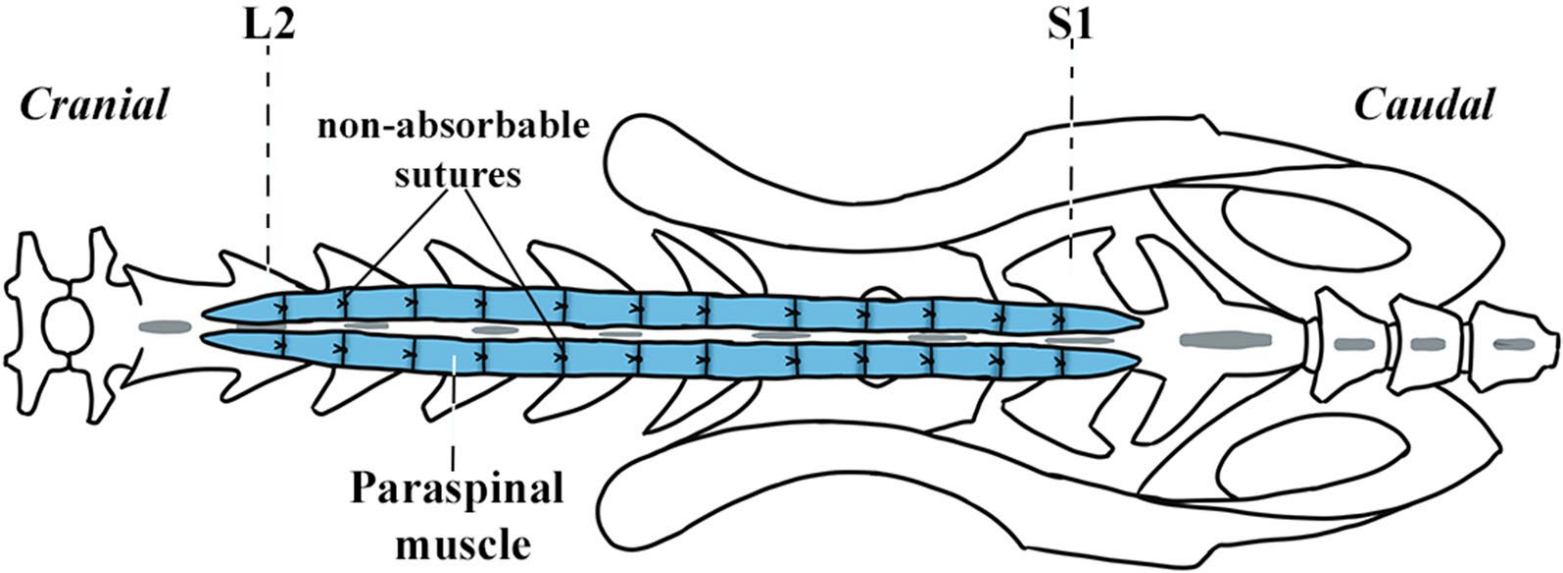
Scheme of the compression model. Rat’s lumbosacral spine with binding of paraspinal muscles (blue) from L2 to S1

The *compression model* (*n* = 5) involved binding the paraspinal muscles from L2 to S1 using non-absorbable sutures made from Nurolon® 3 (Ethicon, USA) as shown in the scheme (Fig. 1) and described in more detail in our previous publication [9].

In the control group, there were five intact animals of a similar age and gender, which were kept on a standard diet without surgery. 90 days after the beginning of the experiment, all 15 rats were euthanized via decapitation under ether anaesthesia.

### Histological analysis

The facet joints and IVDs were taken out from the rats (at the L1–L4 level) along with the adjacent vertebrae. The fragment was divided into two segments: the first segment, L2–L3, was cut in the coronal plane for intervertebral disc investigation, and the second segment, L3–L4, was cut in the sagittal plane for facet joint investigation. The material was fixed for 5 days in neutral 10% formaldehyde, decalcified for 5 days in 5% formic acid, then dehydrated in isopropyl alcohols of growing concentration (from 50 to 100%), and embedded in paraffin. The histological sections were prepared on a Reichert base sledge microtome and stained with hematoxylin and eosin (H&E) and Picrosirius Red/Alcian Blue (P&A) [16]. The sections were analysed under a BX63 (Olympus, Japan) light microscope and photographed using a DP73 (Olympus) digital camera with the “Cell Sens Dimension 1.8.1” software (Olympus, 2013).

Two independent morphologists conducted a semi-quantitative analysis of the structure of the rats’ IVDs and facet joints. Changes in the structure of the cells and matrix in the IVDs were evaluated according to the degeneration grading system for rat intervertebral discs [17]. The articular cartilage of the facet joints was assessed using the Cartilage degeneration scale recommended by the Osteoarthritis Research Society International (OARSI) and standardized for rats. On that scale, a score of 0 corresponds to no degeneration and 5 corresponds to severe degeneration [18]. The average values for the degenerative changes in the rats’ facet joints and IVDs were calculated. On each scale, the tissue with a typical structure had the lowest value, while the most damaged tissue had the largest value.

### Histomorphometry

Morphometric studies of the IVDs and articular cartilage of the facet joints were conducted using the “CellSens Dimension 1.8.1” software (Olympus, 2013). The height (µm) of the IVD was measured on its edges and central part. The thickness (µm) of articular cartilage in the facet joints was also measured (8–10 measurements on each articular surface of each section).

### Statistical analysis

The mean and standard deviation of the obtained values are shown. The normality of the distribution of the results was verified using the Kolmogorov–Smirnov test, and the group means were compared using unpaired Student's *t* test. The difference in values between the groups was statistically significant with *p* < 0.05. The statistical analysis was conducted using the IBM SPSS Statistics 23 software.

## Results

### High-fat diet model

**Fig. 2:**
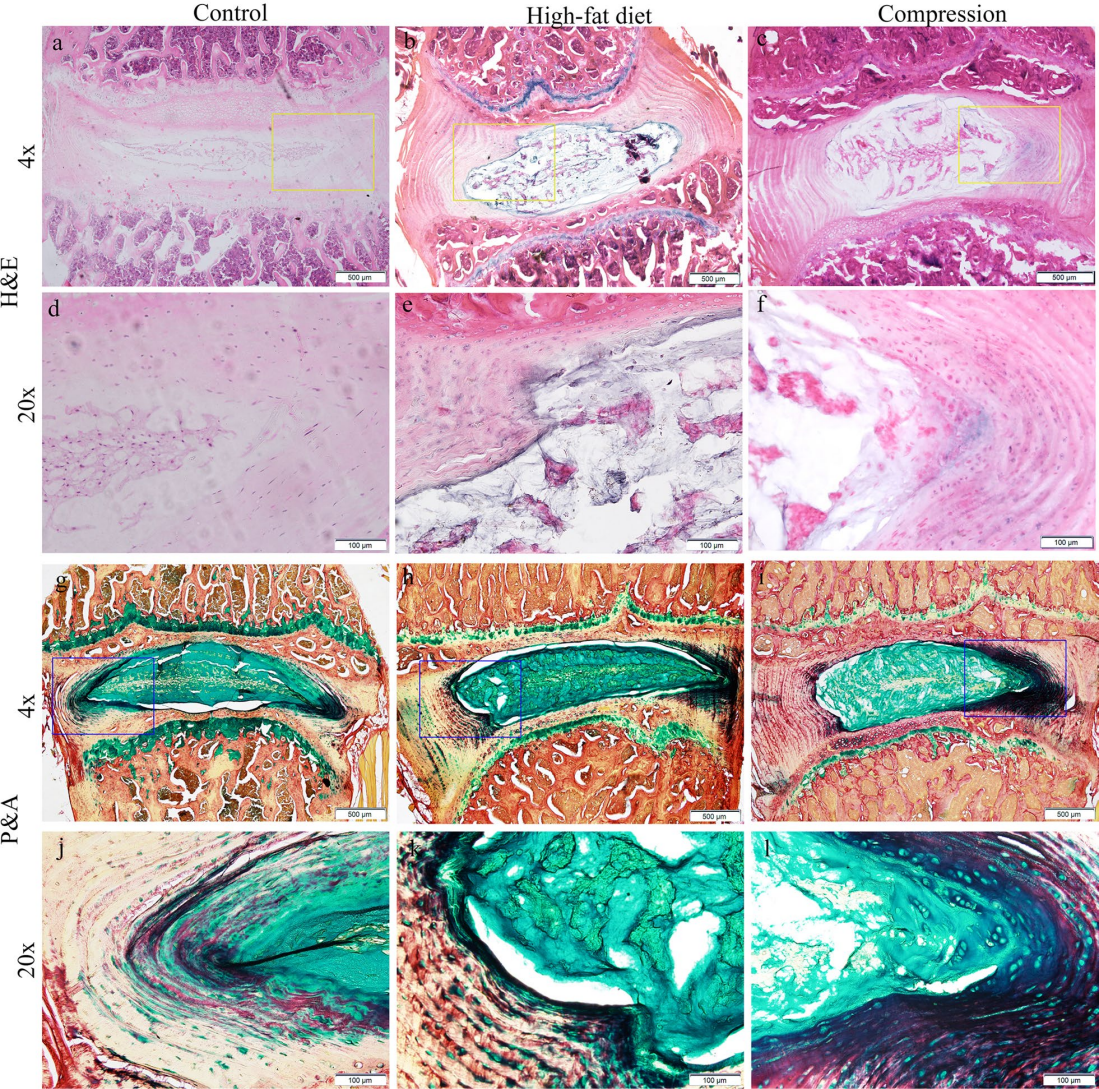
Histological findings in rats’ IVDs L2–L3. Control group, regular structure: **a**, **d** Hematoxylin and Eosin stain (H&E), **g**, **j** Picrosirius Red/Alcian Blue stain (P&A). High-fat diet: **b** fissures and separation in the lamella of the annulus fibrosus, the nucleus pulposus bulging towards the vertebra, structural changes of the subchondral bone; **e** fragment of 2b: the nucleus pulposus bulging towards the vertebra, H&E; **h** decrease in the intensity of the staining of matrices in the inner annulus fibrosus; **k** fragment of 2 h: destructive changes in nucleus pulposus, P&A. Compression: **c** fissures and separation in the lamella of the annulus fibrosus, decrease in the number of cells, and changes in the matrix structure observed in the nucleus pulposus, degenerative changes in the subchondral bone; **f** fragment of 2c: gaps developing in the border between the annulus fibrosus and the nucleus pulposus, H&E; **i** the intensity of the Alcian blue matrix staining (cyan colour) in the inner annulus fibrosus greater than the control; **l** fragment of 2i: the formation of clusters of cells, P&A

After 90 days of keeping the rats on a high-fat diet, moderate degenerative changes were observed in the rats’ IVD: fissures and separation in the lamella of the annulus fibrosus, a reduction in the number of cells in the lamella, a decrease in the intensity of the P&A staining of matrices in the inner annulus fibrosus, in comparison with the control group (Fig. 2a, b, d, e, h, k). Only mild degenerative changes were found in the nucleus pulposus, as opposed to the annulus fibrosus: a decrease in the number of cells and matrix damage were only observed in one rat. Regions of matrix without chondrocytes and microfractures were discovered in the end plates of uneven thickness. In three rats, the nucleus pulposus was bulging towards the vertebra, which can be interpreted as the first step in the formation of a hernia. In that region, a disturbance in the structure of the subchondral bone was observed: thinner trabeculae, significant trabecular areas without osteocytes, and the formation of many destructive fissures (Fig. 2b, e).

**Fig. 3:**
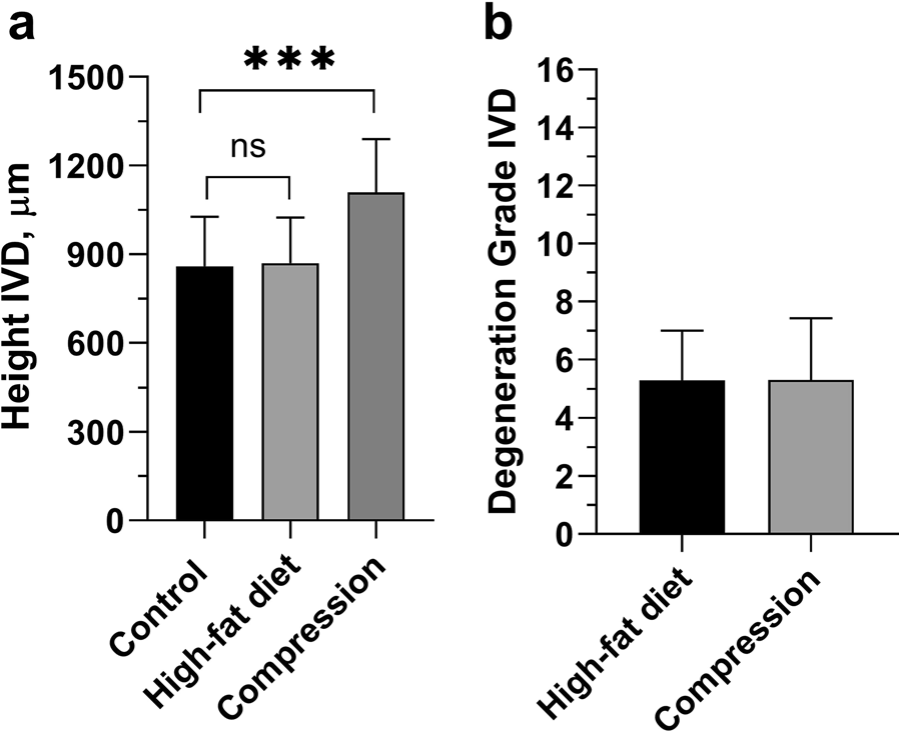
Histomorphometry of rats’ lumbar IVDs: **a** height (µm); **b** degenerative disturbance score (in points) according to the degeneration grading system for rat intervertebral discs. Unpaired Student *t* test. ***p < 0.001

The height of the IVD in rats kept on a high-fat diet did not differ from the control group (869.42 ± 155.03 µm *vs 0.8*58.73 ± 167.93 µm; *p* = 0.546) (Fig. 3). Degenerative changes in the IVD were scored 5.3 ± 1.7 out of a possible 16 (Fig. 3), which corresponds to ≈30% of the disc being damaged.

**Fig. 4:**
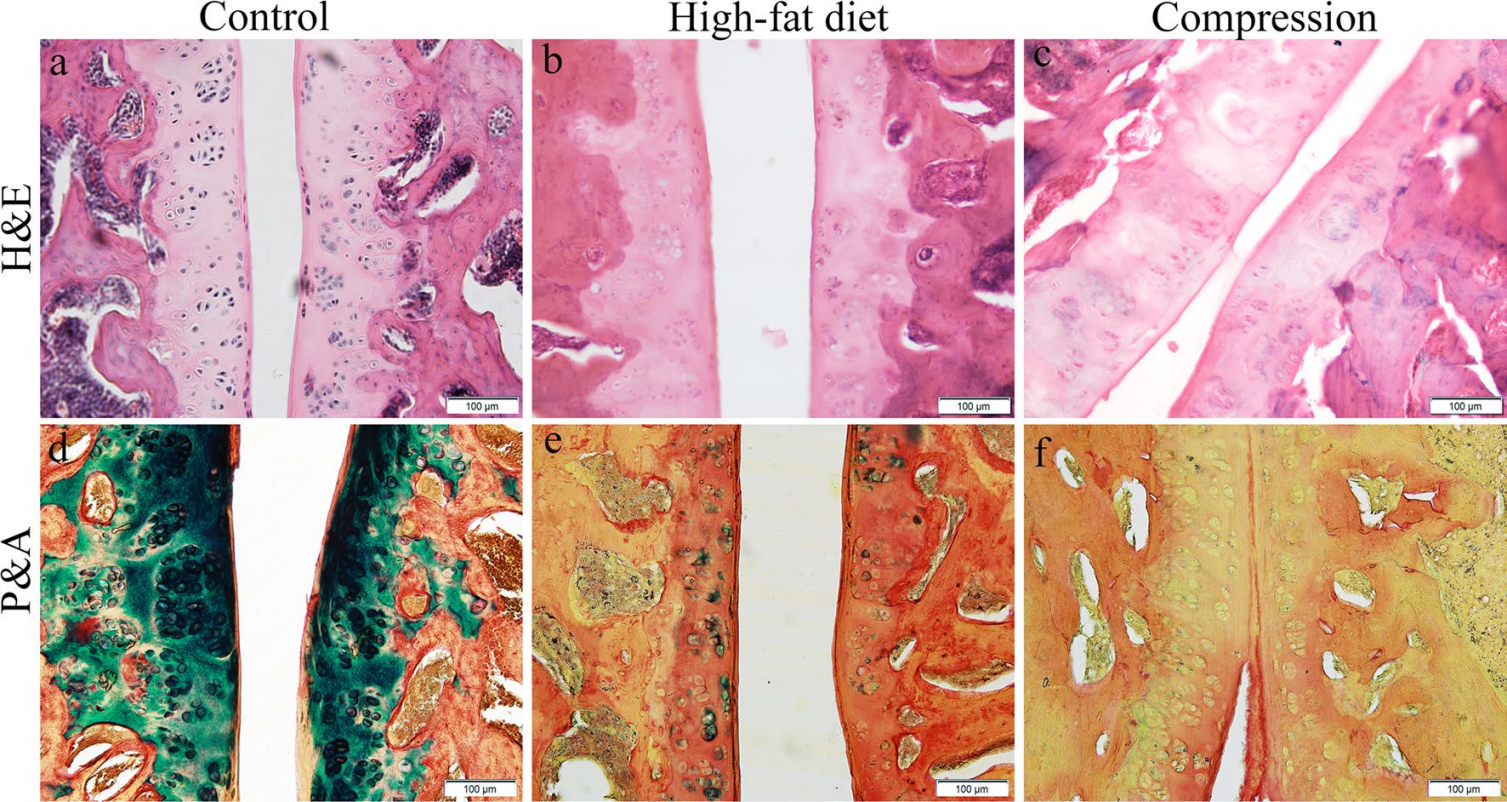
Histological finding in the articular cartilage of the rats’ facet joints L3-L4. High-fat diet: **b** areas without cells, uneven colouring of the matrix, growth of blood vessels into the articular cartilage, and empty lacunae, Hematoxylin and Eosin stain (H&E); **e** intensity of the Alcian blue matrix staining (cyan colour) around these clusters significantly lower than control, Picrosirius Red/Alcian Blue stain (P&A). Compression: **c** marked degenerative changes in the articular cartilage: cell death, necrotic areas, H&E; **f** decrease in glycosaminoglycan concentration (absent of cyan colour) compared to the control, P&A

Marked degeneration was observed in the facet joints: formation of large clusters of chondrocytes (6–30 cells), areas without cells, uneven staining of the matrix, and growth of blood vessels into the articular cartilage. The majority of chondrocytes were hypertrophied. In some clusters, only empty lacunae remained (Fig. 4b). The intensity of the P&A matrix staining around these clusters (cyan colour) was significantly lower compared to the control (Fig. 4e). Hypertrophy of the synovium was also observed. Additionally, an increase in trabeculae density was discovered in the subchondral bone, while the spaces between trabeculae were filled with connective tissue.

**Fig. 5:**
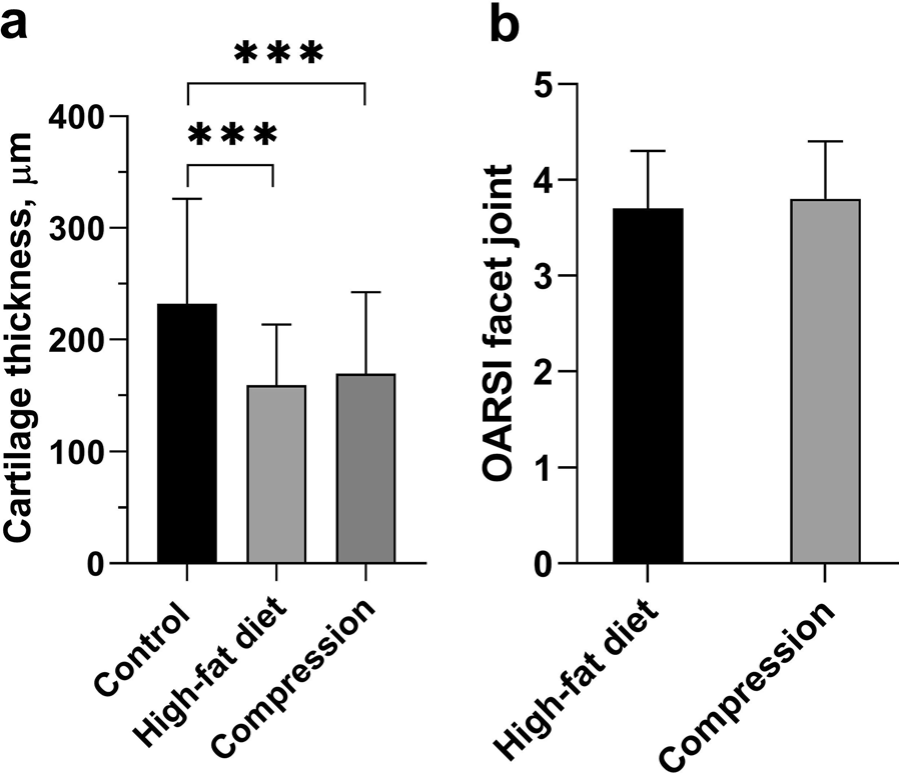
Histomorphometry of rats’ lumbar facet joints: **a** cartilage thickness (µm); **b** degenerative disturbance (in points) according to the OARSI cartilage degeneration score. Unpaired Student *t* test. ***p < 0.001

The height of the articular cartilage of the facet joints in rats kept on a high-fat diet was smaller by 1.5 times (159.01 ± 54.39 µm *vs*. 232.14 ± 94.02 µm; *p* < 0.001) compared to the control. Degenerative changes were scored 3.7 ± 0.6 out of five points (Fig. 5).

### Compression model

Similar to the high-fat diet model, moderate degenerative changes were observed in the IVDs of rats with compressed paraspinal muscles, including fissures and separation in the lamella of the annulus fibrosus, an uneven distribution of cells within the lamella, the lamella of the inner annulus fibrosus taking on a “wavy” shape, and gaps developing in the border between the annulus fibrosus and the nucleus pulposus (Fig. 2c, f). The intensity of the P&A matrix staining in the inner annulus fibrosus (cyan colour) was greater than the control (Fig. 2i, l). Furthermore, intra-tissue swelling was observed. A decrease in the number of cells (less than ^2/3^ of the original area occupied) and a disturbance in the matrix structure were observed in the nucleus pulposus (Fig. 2c, f). The formation of clusters of cells can be seen as a result of the P&A staining (Fig. 2l). In addition to the changes described in the high-fat diet model, calcification and osteophyte formation were observed in the end plate.

As opposed to the high-fat diet model, the height of the IVD was 1.3 times greater (1108.28 ± 181.61 µm vs. 858.73 ± 167.93 µm; *p* < 0.001) in the compression model when compared to the control (Fig. 3). Degenerative changes in the IVD were scored 5.32 ± 2.1 out of a possible 16 points, which, similarly to the high-fat diet model, corresponds to ≈30% of the disc being damaged.

Marked degenerative changes were observed in the facet joints. Large clusters of chondrocytes (6–30 cells), some of which were only composed of empty lacunae, were found in the middle zone of the articular cartilage. In addition, significant areas without cells, uneven staining of the matrix, and necrotic areas were observed in that zone (Fig. 4c). P&A staining confirmed a decrease in glycosaminoglycan content (cyan colour) compared to the control (Fig. 4f). Also observed were a narrower joint cavity, and superficial and deeper fibrillation of the articular cartilage. The synovium was hypertrophied. Similar to rats in the high-fat diet model, the density of bone trabeculae increased in the subchondral bone, while bone marrow was replaced with connective tissue.

The height of the articular cartilage of the facet joints was 1.4 times (169.51 ± 72.95 µm *vs.* 232.14 ± 94.02 µm; *p* < 0.001) smaller compared to the control (Fig. 5). Degenerative changes in the joints were scored 3.8 ± 0.6 out of five points according to the cartilage degeneration score (Fig. 5).

## Discussion

This study is a continuation of our previously published work, where we demonstrated the development of degenerative changes in the paraspinal muscles that occur when rats are kept on a high-fat diet for 90 days or due to lumbar paraspinal muscle compression [9]. It was determined that after the high-fat diet, the areas of fat in the m. multifidus and m. psoas were, respectively, 1.8 and 2.2 times higher than in the control group. Prolonged muscle compression resulted in degenerative changes in the lumbar m. multifidus, such as the formation of fat and fibrous tissue (12.66% of the total area).

The results of the current study indicate that both in vivo models create moderate degenerative changes in the IVDs and facet joints. Although the high-fat diet model is a systemic model, while the compression model is a local model, the degenerative changes observed in the studied structures of the spine were very similar.

In both models, 90 days after the experiment, the degenerative changes observed in the rats’ IVDs were more severe in the annulus fibrosus than in the nucleus pulposus. The end plates did not have an even thickness, cells were unevenly distributed, and fissures were discovered. According to the degeneration grading system for rat intervertebral discs, the level of disturbance was around 30%. As opposed to the high-fat diet model, the height of the IVD was 1.3 times greater (*p* < 0.001) in the compression group when compared to the control, which is probably caused by its swelling. Moreover, P&A staining was used to demonstrate an increase in the content of glycosaminoglycans in the inner annulus fibrosus in the compression model, as compared to the control and high-fat diet model, which can be explained by a partial regeneration of the cartilaginous tissue [19]. Similar to our results, Iatridis et al. [20] demonstrated an elevated proteoglycan content in the intervertebral disc after compressing the rat tail using an Ilizarov-type apparatus. Additionally, the increase in glycosaminoglycan (proteoglycan) content may contribute to a higher fluid content in the disc, which probably explains the increased disc height in the compression model. At the same time, a decrease in the intensity of P&A staining (cyan colour) found in the rats kept on a high-fat diet is similar to the degenerative changes of the matrix, which was described in the anterior lumbar disc needle puncture model [21].

It was clinically proven that obesity is a predictor of lumbar spine degeneration and LBP [22], and that body mass index correlates with the severity of the degeneration of the IVD [23]. Degenerative changes were observed in the IVDs of rats kept on a high-fat diet, which could be connected to a systemic failure in the nutrition of the paraspinal muscles and their degeneration, as their contraction ensures proper venous function and blood flow to structures of the spine. Additionally, the contraction of the paraspinal muscles, along with the stretching of the tendons, is crucial for the proper distribution of load on the IVD [24]. Hypothetically, the load on the lumbar IVDs in large quadrupeds, such as cows or pigs, may even exceed the load on the lumbar IVDs in humans because of the higher forces required to stabilize a horizontal spine as opposed to a vertically-oriented one [25, 26]. Although rat spines experience much lower loads than those of humans, there is still a belief that the intradiscal pressure may be similar between the two [25, 26]. Since compressing muscles in the lumbar spine changes the distribution of mechanical load, this can influence the intradiscal pressure. The accumulation of fat in muscles at the L4–L5 level is associated not only with pain, but also with a disturbance in the structure of the end plate (Type 1 and 1/2 Modic changes) [27].

In the compression model, the rats did not undergo mechanical trauma or excessive load and were not treated with chemical agents. Therefore, we can assume that the mechanism of the development of degenerative changes in the IVDs may be connected to their improper nutrition caused by the local compression of blood vessels in the paraspinal muscles, which was created by binding them with non-absorbable sutures. For example, a decrease in the intake of nutrients due to locally calcified blood vessels can cause degeneration in the IVD [28].

In both models, moderate degenerative changes (according to the OARSI cartilage degeneration score) were observed in facet joints. Clusters of chondrocytes (6–30 cells), large areas without cells, empty lacunae, and blood vessel growth were observed in the middle zone of the articular cartilage. Furthermore, a decrease in the concentration of glycosaminoglycans was found, which is evidence of reduced biosynthesis in the chondrocytes [29]. The height of the articular cartilage in the facet joints was smaller in both the high-fat diet and compression models than in the control (1.5 and 1.4 times, respectively). The joint cavity was narrower and the synovium was hypertrophied. These changes correspond to the development of osteoarthritis in the facet joints. Some authors believe that the observed changes in the subchondral bone, such as increased bone trabeculae density and morphological changes in the bone marrow, are the main histopathological features of osteoarthritis in the facet joints [30].

Similar pathological changes in the rats’ facet joints can be obtained by intraarticular injection of chemical agents [31, 32]. This also leads to the development of pain syndrome due to synovitis and osteoarthritis in the facet joints. The mechanism of development of pathological changes in these models is explained by the direct effect of the chemical agents on the metabolism of the chondrocytes [31], or the ability of these chemical agents to cause lysis in the intercellular matrix and basal membrane [32]. However, these studies did not take into account the resection or retraction of the m. multifidus, which were conducted in order to access the facet joints. Another model of osteoarthritis in rats’ facet joints is the implantation of compression springs, fixed between the spinous processes to ensure a constant compression force parallel to the spinal axis [33].

In our study, osteoarthritis in rats’ facet joints was developed by keeping rats on a high-fat diet or compressing the paraspinal muscles for 90 days. One of the probable mechanisms of the development of osteoarthritis in the high-fat diet model is the disturbance of the nutrition and biomechanics of the locomotion segments in the lumbar spine due to the accumulation of fat in the paraspinal muscles [9]. In the compression model, the development of osteoarthritis in the facet joints and degeneration in the IVDs is also connected to changes in their nutrition. In this scenario, the changes occur due to the binding of paraspinal muscles and compression of the blood vessels. A strong correlation between the stage of abdominal aortic calcification and the development of osteoarthritis in the facet joints was established clinically, which further illustrates the importance of blood circulation for proper spinal function [34].

The obtained results make it possible to expand knowledge about the occurrence of degenerative changes in the lumbar spine in obese individuals, which arise not only due to the action of a mechanical factor (excess body weight) but also due to the action of chemical factors (pro-inflammatory cytokines, etc.) associated with obesity. As a result of the compression model study, it was shown that local fatty dystrophy of the paraspinal muscles causes degenerative changes in the lumbar spine. This confirms the need for an active lifestyle and the creation of rehabilitation programmes, including after spinal surgery.

## Conclusions

It was determined that rats who had structural changes in the lumbar paraspinal muscles as a result of being kept on a high-fat diet or subjected to prolonged compression for 90 days, showed degenerative changes in the lumbar intervertebral discs and osteoarthritis in the lumbar facet joints. The degeneration of paraspinal muscles can be considered one of the etiological factors in the development of degenerative spine diseases.

## Data Availability

The datasets used and analysed during the current study are available from the corresponding author on reasonable request.

## Abbreviations

H&E: Hematoxylin and eosin
IVD: Intervertebral disc
LBP: Low back pain
OARSI: Osteoarthritis research society international
P&A: Picrosirius red and Alcian blue
VAS: Visual analogue scale
